# Updates and Controversies in the Rapidly Evolving Field of Lung Cancer Screening, Early Detection, and Chemoprevention

**DOI:** 10.3390/cancers6021157

**Published:** 2014-05-16

**Authors:** Hasmeena Kathuria, Yaron Gesthalter, Avrum Spira, Jerome S. Brody, Katrina Steiling

**Affiliations:** 1 The Pulmonary Center, Boston University School of Medicine, 72 East Concord Street, Boston, MA 02118, USA; E-Mails: yaron.gesthalter@bmc.org (Y.G.); jbrody@bu.edu (J.S.B.); 2 Division of Pulmonary, Allergy, and Critical Care Medicine, 72 East Concord Street, Boston University School of Medicine, Boston, MA 02118, USA; 3 Division of Computational Biomedicine, Boston University School of Medicine, 72 East Concord Street, Boston, MA 02118, USA; E-Mails: aspira@bu.edu (A.S.); steiling@bu.edu (K.S.); 4 Bioinformatics Program, Boston University, 24 Cummington Mall, Boston, MA 02215, USA; 5 Department of Pathology and Laboratory Medicine, Boston University School of Medicine, 72 East Concord Street, Boston, MA 02118, USA

**Keywords:** lung cancer, screening, early detection, chemoprevention

## Abstract

Lung cancer remains the leading cause of cancer-related death in the United States. Cigarette smoking is a well-recognized risk factor for lung cancer, and a sustained elevation of lung cancer risk persists even after smoking cessation. Despite identifiable risk factors, there has been minimal improvement in mortality for patients with lung cancer primarily stemming from diagnosis at a late stage when there are few effective therapeutic options. Early detection of lung cancer and effective screening of high-risk individuals may help improve lung cancer mortality. While low dose computerized tomography (LDCT) screening of high risk smokers has been shown to reduce lung cancer mortality, the high rates of false positives and potential for over-diagnosis have raised questions on how to best implement lung cancer screening. The rapidly evolving field of lung cancer screening and early-detection biomarkers may ultimately improve the ability to diagnose lung cancer in its early stages, identify smokers at highest-risk for this disease, and target chemoprevention strategies. This review aims to provide an overview of the opportunities and challenges related to lung cancer screening, the field of biomarker development for early lung cancer detection, and the future of lung cancer chemoprevention.

## 1. Introduction

Lung cancer is the leading cause of cancer mortality, accounting for almost 27% of all cancer-related deaths [1] and 20% of total U.S. Medicare expenditures for cancer [2]. Survival rates for newly diagnosed lung cancer remain at approximately 17% [3], minimally improved over the past three decades, largely because the disease is most often diagnosed at an advanced stage when there are few curative treatment options. Patients with early-stage lung cancer have improved survival compared to those with late stage disease, suggesting that early detection could improve mortality from this disease. An estimated 85% of lung cancer cases in the United States are caused by cigarette smoking [4]. Although smoking incidence is projected to plateau at 20% of the United States adult population by 2030, the persistent risk of lung cancer in former smokers suggests that lung cancer will remain a major health problem for years to come.

This review will provide an overview of the progress that has been made in lung cancer screening, early detection, chemoprevention, and biomarker development using surrogate tissues. It will also discuss some of the progress and controversies in these areas, and how evolving technologies may improve our ability to target interventions for the subpopulations at highest risk for lung cancer. The prime focus of this review is on smoking-associated lung cancer since cigarette smoking is a major contributor to development of this disease.

## 2. Quantifying Lung Cancer Risk

Although 80%–85% of patients with lung cancer have a history of smoking, only 10%–15% of patients who smoke will actually develop lung cancer. There is presently no widely-accepted method to accurately predict which current and former smokers will develop lung cancer. The risk of developing lung cancer increases with accumulated exposure to cigarette smoke, which alters antioxidant, xenobiotic, inflammatory-related pathways and oncogenic genes. Identifying the subset of cigarette smokers at highest risk for developing lung cancer would enhance the ability to target lung cancer surveillance, chemoprevention, and early detection strategies. Furthermore, risk modeling can facilitate the design of targeted clinical trials by enrolling higher risk subjects.

Although cigarette smoking is the major risk factor for lung cancer, lung cancer also occurs at lower rates in nonsmokers (individuals who have had a lifetime exposure of fewer than 100 cigarettes). Wakelee *et al.* estimated a lung cancer incidence in never-smokers age 40–79 years ranging from 4.8–13.7 per 100,000 person-years in men and 14.4–20.8 per 100,000 person-years in women [5]. Thu *et al.* showed that non-smoker’s lung cancer had a greater portion of their genome altered compared to smokers lung cancer [6]. These observations raise the question of whether lung cancer in never-smokers might result from unrecognized environmental or toxic exposures, especially in genetically susceptible individuals.

Bach *et al.* were the first to apply modern statistical methods to assess 10-year lung cancer risk in heavy smokers by considering age, sex, smoking history, and asbestos exposure [7]. This model, however, underestimates lung cancer mortality for subjects followed for longer durations [8]. Spitz *et al.* developed another risk assessment model that incorporated clinical information to predict 1-year lung cancer risk in current, former, and never smokers [9]. The Liverpool Lung Project (LLP) modelled 5-year lung cancer risk by using information on smoking duration, history of pneumonia, asbestos exposure and prior malignancy diagnosis [10]. However, the difficulty in achieving clinically useful risk-assessment models was illustrated in a comparison of these three models, which demonstrated high positive predictive values but low negative predictive values for identifying individuals at high risk for lung cancer [11]. Incorporating additional data, such as socio-demographic information, early clinical symptoms, and radiographic findings may improve model performance [12]. For example, Maisonneuve and colleagues used data from the COSMOS lung cancer screening trial to modify the Bach risk model to incorporate additional radiographic variables [13]. These observations suggest that risk assessment models can identify individuals at high risk for developing lung cancer, but to date no risk model for lung cancer has shown an improvement in survival in trials.

Incorporating molecular information with clinical risk features may improve the ability to identify individuals with the highest risk for developing lung cancer. For example, it is well established that smokers who are first-degree relatives of individuals with lung cancer have a two- to three-fold higher risk of developing lung cancer themselves [14]. Polymorphic variants in almost 50 genes have been associated with alterations in lung cancer risk [15], and could potentially be incorporated to further refine lung cancer risk models. For example, risk models of lung cancer that incorporate single nucleotide polymorphisms have been shown to distinguish lung cancer cases from normal controls without lung cancer. Such models may improve targeted prevention and early diagnostic strategies in lung cancer [16,17]. Other molecular information, such as epigenetic and gene or microRNA expression profiling, might also be used to refine risk prediction models. Spitz *et al.* expanded their clinical risk model for current and former smokers to include two markers of DNA repair with marginal improvement in discriminatory power of the model [18]. While such hybrid models incorporating both clinical and molecular risk factors are promising, they remain to be validated in prospective clinical trials.

## 3. Imaging-Based Lung Cancer Screening

**Figure 1:**
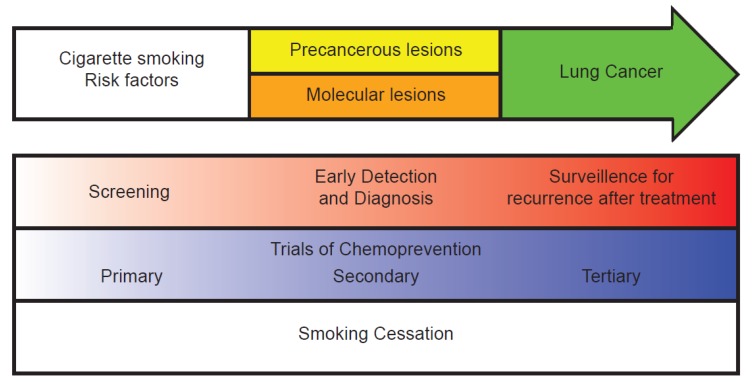
Lung cancer screening, early detection, and chemoprevention. While cigarette smoking is the major risk for lung cancer, other risk factors such as toxic exposure including asbestos and a family history of lung cancer influence lung cancer risk. In some individuals susceptible to lung cancer, molecular abnormalities or pre-cancerous dysplastic lesions develop. A subset of these individuals will ultimately progress to lung cancer. Because survival from lung cancer is lower when the disease is diagnosed at an advanced stage, screening high-risk individuals or developing early detection strategies will improve mortality. Although clinical trials have not demonstrated a benefit to chemoprevention, the potential to develop targeted risk-assessment and preventive strategies at all stages of clinical and pre-clinical risk exist. Smoking cessation should be an integral part of all stages of lung cancer screening and treatment.

The drastic drop in survival rates with delayed diagnosis of lung cancer demonstrates the pressing need for effective screening strategies. Ideally, a screening tool would accurately identify early stage lung cancer patients in a safe and cost effective manner (Figure 1). Furthermore, an ideal lung cancer screening test would have high specificity, limiting unnecessary invasive procedures and costly follow up studies [19]. However, given the heavy disease burden and enormous personal and public health consequences of lung cancer, even a small benefit from screening could save many lives. 

Previous lung cancer screening trials using chest radiography [20,21] and/or sputum cytology [22] did not demonstrate an effect on lung cancer mortality [23,24]. The Mayo Lung Project investigated the benefit of annual chest radiography and sputum cytology by randomizing over 9000 male smokers to these interventions or usual care. Even during extended follow-up, there was no improvement in mortality [22]. The Prostate, Lung, Colorectal and Ovarian (PLCO) Cancer Screening trial was one of the largest trials to evaluate the utility of chest radiography for lung cancer screening. Over 154,000 participants aged 55 to 74 were randomly assigned to receive annual chest X-ray for four consecutive years or usual care [21]. Annual screening with chest radiograph did not reduce lung cancer mortality compared with usual care, thus confirming results from previous screening trials. 

Computerized tomography (CT) has also been investigated as a lung cancer screening tool given its improved ability to radiographically evaluate other lung diseases compared to chest radiography. While smaller and observational CT screening studies did not show a mortality benefit, the large randomized National Lung Screening Trial (NLST) did demonstrate a statistically significant benefit to using annual low-dose CT (LDCT) screening in individuals at high risk for lung cancer. This randomized trial involved greater than 53,000 current and former heavy smokers (>30 pack-years) compared to annual low-dose helical CT with chest radiography for lung cancer screening. High-risk individuals were defined as men and women aged 55 to 74 who were active or former (<15 years since quitting) smokers with a cumulative exposure to cigarettes of at least 30 pack-years. A significant improvement in mortality from lung cancer and all causes was observed for LDCT screening (20% and 6.7% respectively), with a number needed to screen of 320 [25]. Based on this data, the U.S. Preventive Services Task Force has given a Grade B recommendation for annual LDCT screening of high-risk smokers (aged 55–80, 30 pack-year cumulative smoking exposure, current smokers or former smokers who have stopped smoking within the past 15 years) [26].

Despite the improvement in mortality with LDCT screening, a high proportion of false positives is observed with this technique. This raises questions on how best to implement LDCT screening in clinical practice, and additional trials are aimed at further evaluating the role of LDCT in lung cancer screening. The randomized NELSON trial in The Netherlands and Belgium is comparing LDCT to no intervention to determine whether LDCT improves lung cancer mortality [27]. This trial will also assess whether a nodule management protocol based on volumetry and volume doubling time (VDT) reduces the false-positive rate associated with LDCT screening [28]. Results from this trial are expected in 2015. The Danish Lung Cancer Screening Trial (DLCST), a single center randomized control trial of 4104 men and women aged 50–70 years old comparing LDCT to no screening intervention, did not show improvement in lung cancer or all-cause mortality after five annual LDCTs [29]. The data from DLCST and other smaller European studies will likely be pooled for combined analysis in order to complement available data on risk populations [29,30]. 

Despite the mortality benefit for high-risk smokers screened with LDCT, the debate over safety and cost effectiveness of this screening protocol is ongoing. Most notably, 23.3% of LDCT screening tests performed in the NLST had false positive results [25]. The potential impact of such a high rate of false positives, such as financial cost, potential complications from additional diagnostic evaluation including biopsy or thoracic surgery, and anxiety associated with diagnostic uncertainty [31,32], is not insignificant when applied to a large-scale screening program. This highlights the importance of improving the benefit to risk ratio in lung cancer screening [33]. To begin to address this question, Kovalchik *et al.* stratified participants in the NLST into five groups based on their risk of developing lung cancer based on clinical factors. Individuals with the highest lung cancer risk benefited most from LDCT screening, suggesting that targeting highest-risk individuals and incorporating lung cancer risk models into CT-screening programs may ultimately improve the benefit and reduce the potential harm and cost from screening [34]. 

Another potential harm of annual LDCT screening is the inevitable exposure to radiation. This is particularly important to consider given the high proportion of patients that will require additional follow up imaging for indeterminate nodules, further increasing cumulative radiation exposure [35]. It has been suggested that one in every 2500 persons screened will develop a radiation-induced lung cancer [36]. While this would likely be more of a factor in younger patients who would endure longer periods of screening compounded by the synergistic oncogenic effects of smoking and radiation [36,37], it emphasizes the need to further refine screening protocols to identify the patients most likely to benefit.

The financial implications of annual LDCT screening are unclear, and this will likely be closely monitored as additional data becomes available. The rate of over diagnosis and false positives may strongly influence the ultimate economic burden of any large-scale lung cancer-screening program, particularly when compared to the cost effectiveness of smoking cessation [38]. Furthermore, it is possible that some of the lung cancers detected by LDCT screening may be indolent. In one study, the magnitude of over-diagnosis in the NLST was estimated to be 18.5% [39]. Taken together, these issues will shape the ongoing discussion of how to best implement lung cancer screening on a large-scale basis in order to reduce lung cancer mortality and minimize harm, while emphasizing the importance of developing additional models in order to refine screening algorithms. 

## 4. Early Detection Biomarkers for Lung Cancer

While the overall 5-year survival for non-small cell lung cancer (NSCLC) is 14%, survival for Stage I disease is 83% [40]. This highlights the importance of detecting lung cancer at an early and potentially treatable stage. In addition to detection of lung cancer through screening protocols, developing biomarkers that are highly sensitive and specific may also improve mortality by identifying individuals with early stages of disease. Early diagnostic biomarkers for lung cancer often use surrogate tissues that do not require sampling of lung tissue itself, and generally aim to detect the presence of an early stage lung cancer or differentiate an early stage lung cancer from a similar-appearing benign pulmonary nodule (Figure 1). 

### 4.1. Bronchial Airway Biomarkers

Bronchial airway biomarkers for early lung cancer detection are based on the concept of “field cancerization” which stems from the observation that some alterations found in the lung tumor are also present throughout uninvolved areas of the respiratory tract and lung. This field cancerization effect, measurable throughout the respiratory tract, is thought to stem from: (1) an airway-wide response to the toxins in cigarette smoke that reflect an individual’s risk for lung cancer; (2) response of the lung and respiratory tract to the presence of tumor; and/or (3) clonal-expansion of the tumor [34]. Prior studies have identified similar mutations in lung tumors adjacent noncancerous lung tissue, and cytologically normal bronchial epithelium [41,42,43,44]. Similarly, Powell *et al.* identified loss of heterozygosity in bronchial brushings obtained from patients with lung cancer [45], suggesting that sampling of sites proximal from the lung tumor might be used to develop diagnostic tools for lung cancer. 

Subsequent studies have leveraged this field cancerization concept to develop clinical-useful lung cancer diagnostics. Using cytologically normal large-airway epithelial cells obtained at bronchoscopy from active and former smokers undergoing clinical evaluation for suspected lung cancer, Spira and colleagues developed an 80-gene expression biomarker that distinguis0hed individuals with and without lung cancer. This airway gene-expression biomarker had an accuracy of 83% (80% sensitivity, 84% specificity) in an independent test set, and ~90% sensitivity for Stage I lung cancer across all subjects, suggesting utility as an early diagnostic tool [46]. Furthermore, the biomarker performed independently from other clinical features, suggesting that incorporating molecular and clinical factors can improve the ability to diagnose lung cancer at an early stage [47]. Molecular analysis of bronchoalveolar lavage fluid may also have utility for developing lung cancer biomarkers as demonstrated in a glycoproteomic study of individuals with and without lung cancer [48]. Other groups have used profiling of genetic variants, methylation, and proteins to identify lung cancer-associated profiles in bronchial airway samples (reviewed in [49]), although all of these biomarkers remain to be validated in prospective clinical trials. Bronchoscopy-based biomarkers may additionally be useful in the evaluation of individuals with indeterminate pulmonary nodules detected by CT-screening. 

Observations that cigarette smoke-induced alterations in the bronchial airway epithelium [50,51] are similarly altered in epithelial cells lining the nose and mouth [52,53] has led to the hypothesis that upper respiratory tract sampling might also be used to develop early diagnostic biomarkers, which could be applied on a larger scale due to their minimally invasive nature. Boyle and colleagues evaluated whole-genome gene expression profiling of punch biopsies from the buccal mucosa of 40 healthy smokers and 40 healthy non-smokers, and identified a strong association between the smoking-induced gene-expression changes in the buccal and bronchial epithelium [54]. Together, these observations point towards the utility of sampling the upper airway epithelium of the nose and mouth to develop clinically-useful early diagnostic biomarkers for lung cancer, and potentially biomarkers to assess lung cancer risk.

### 4.2. Sputum Biomarkers

Another potential surrogate tissue for developing early diagnostic biomarkers for lung cancer is sputum. Despite sputum cytology not being an effective screening method to achieve early lung cancer diagnosis [55], subsequent studies have shown that mutations associated with lung cancer, such as in Kras and p53, are detectable in the sputum of patients with this disease [56]. Deletions in HYAL2 and FHIT, present in lung tumors, have been shown to be significantly concordant with deletions in paired sputum samples and significantly higher in patients with lung cancer compared to smokers without lung cancer [57]. Fluorescent *in situ* hybridization (FISH) can be used to detect genetic changes in sputum and microRNA panels, identified by profiling primary lung tumors and adjacent noncancerous lung tissue, and have been measured in sputum as a potentially useful tool for early lung cancer detection [40]. 

Furthermore, DNA methylation, an epigenetic event that affects cell function by altering gene expression, has been shown to be altered in sputum samples from patients with lung cancer. Belinsky *et al.*, for example, have shown that hypermethylation of p16 and MGMT are detectable in the sputum of patients with squamous cell lung cancer, and could potentially serve as an early diagnostic for lung cancer [58]. In addition, aberrant methylation profiles have been detected in the sputum of patients with lung cancer three years prior to clinical diagnosis [59]. Some of these events, such as p16 and DAP kinase hypermethylation, are detectable both in the bronchial epithelium and sputum of current and former smokers [58].

### 4.3. Blood-Based Biomarkers

Whole blood, serum and plasma are other easily obtainable surrogate tissues that could be used to develop biomarkers for early lung cancer detection. The source of these biomarkers could be from the tumor itself, or from the host response to the presence of a lung tumor. Serum measurement of the protein pentaxin 3 has been described as a potential early lung cancer diagnostic [60]. Another study showed that complement activation factor c4d, identified in a comparison of human lung cancer cell lines, was higher in the serum of patients with lung cancer compared to those without lung cancer, regardless of stage and was associated with shorter survival [61]. Recently, Boeri and colleagues identified miRNA signatures in plasma samples collected 1–2 years before disease onset that may be useful for predicting lung cancer development and prognosis [62], and Bianchi *et al.* identified a group of miRNA in serum samples that might serve as an early diagnostic biomarker in asymptomatic patients [63]. Other studies have also identified distinct patterns of methylation that distinguish lung adenocarcinoma from normal lung tissue [64,65].

Ostroff *et al.* developed a series of potential biomarkers for early lung cancer diagnosis using a proteomic profiling of serum samples [66]. Other groups have reported circulating tumor cells that could potentially be used to develop biomarkers for early lung cancer detection, but the AUCs were not optimal [67]. Recently, a serum proteomic signature [68] added diagnostic value to established clinical and radiologic parameters routinely used to evaluate indeterminate pulmonary nodules detected by CT [69]. Importantly, a recent study identified a 13-protein biomarker capable of distinguishing benign from malignant nodules detected on CT scan with a high negative predictive value [70]. Together, these studies suggest a potential role for blood biomarkers in the evaluation of indeterminate pulmonary nodules. Although promising, none of these studies have yet tested the diagnostic potential in prospective multi-center trials. 

## 5. Risk Reduction and Chemoprevention

### 5.1. Smoking Cessation

Before the manufacturing of cigarettes became widespread in the early 20th century, lung cancer was a rare disease. Today, cigarette smoking remains a leading cause of lung cancer, with 85% of lung cancer cases attributable to this exposure [71]. 20% of the adult US population however, continues to smoke [72]. Smoking cessation is the only intervention shown to reduce the risk of lung cancer. In the Lung Health Study, smokers were randomly assigned to smoking cessation programs or no intervention [73]. Participants who successfully quit smoking had a 55% reduction in lung cancer incidence compared to the control group. The incorporation of smoking cessation interventions into lung cancer screening programs may also increase the cost effectiveness of screening [74], perhaps by decreasing the continued damage induced by cigarette smoke (Figure 1). 

### 5.2. Chemoprevention

Smoking prevention and cessation are the foundation of any strategy for preventing lung cancer (Figure 1). However, even after people stop smoking, there is an increased risk of developing lung cancer that persists for decades. Chemoprevention, as first defined by Sporn, is the use of naturally occurring or synthetic agents to reverse, suppress or prevent cancer development or progression [75]. Chemoprevention has assumed increasing importance over the last 20 years, as former smokers now account for 50% of new lung cancer cases in the United States. Unfortunately, trials of primary (preventing cancer in healthy individuals who are at high risk), secondary (blocking the development of cancer in individuals with a precancerous lesion), and tertiary (targeting patients with a previous tumor in an effort to prevent the development of a second tumor) chemoprevention agents have not demonstrated consistent benefit in preventing lung cancer in any of these settings. However, given the improved understanding of molecular mechanisms underlying lung carcinogenesis and the elucidation of putative molecular targets, there has been renewed interest in this area of research. Furthermore, successes in chemoprevention of breast, prostate and colon cancer as well as the availability of new FDA-approved agents for the treatment of precancerous lesions has further stimulated interest in lung cancer chemoprevention. 

### 5.3. Clinical Trials

Despite numerous trials, no chemoprevention studies to date have demonstrated an improvement in lung cancer mortality, and few studies have demonstrated an effect on intermediate endpoints such as genomic markers of epithelial cell damage and sputum atypia. Several publications review the results of randomized trials and the rationale for evaluating new chemopreventive agents [76,77,78,79,80,81]. Broad categories of chemopreventive agents and clinical trials of these agents in the prevention of lung cancer are reviewed below and in Table 1. Current guidelines do not endorse the routine use of any compounds for the chemoprevention of lung cancer.

**Table 1:** Summary of major lung cancer chemoprevention agents and trials. Based on the results of existing clinical studies, routine chemoprevention for lung cancer is not currently recommended.

Chemoprevention group	Antioxidants	Anti-inflammatories	PI3K/AKT/mTOR Pathway inhibitors
Primary chemoprevention Healthy patients at high risk for lung cancer	α-Tocopherol [82,83,84] β-Carotene [82,83,84,85,86,87] Selenium [88,89,90,91]	NSAIDs [92,93,94,95] PPARγ agonists [96,97,98]	
Secondary chemoprevention Patients with pre-cancerous lesions	Retinoids [99,100,101,102] Retinoids + β-carotene [103] ADT [104]	Celecoxib [105,106] Iloprost [107]	Myoinositol [108,109]
Tertiary chemoprevention Patients with a previous lung cancer that has been treated	Retinoids [110,111] Selenium [112]		

### 5.4. Primary Chemoprevention

#### 5.4.1. Antioxidants

Based on preclinical studies providing a rationale to study α-tocopherol (vitamin E) and β-carotene, one of the earliest chemoprevention trials evaluated these agents for the primary prevention of lung cancer in smokers as part of the α-tocopherol, β-carotene (ATBC) Cancer Prevention Study. This trial showed an 18% increased incidence of lung cancers and an 8% increased overall mortality for those on β-carotene at a median follow-up of 6 years [82]. In a subsequent analysis, the adverse effects were observed to be stronger in men with a modest alcohol intake and smokers of 20 cigarettes daily [85]. Once supplements were discontinued, the excess risk of lung cancer and death declined [86]. Treatment with α-tocopherol had no effect on the lung cancer incidence or death rate [74]. β-carotene has also been studied in high-risk individuals in the β-Carotene and Retinol Efficacy Trial (CARET) [87]. This study was closed early because those in the treatment arm had a 28% higher rate of lung cancer and a 17% higher overall death rate. Two other randomized studies, the Physicians Health Study [84] and the Women’s Health Study [83], failed to confirm a benefit from either agent. 

Selenium, a component of the antioxidant enzymes glutathione peroxidase and thioredoxin reductase, is thought to improve cellular defense against oxidative stress and has also been studied as a potential primary chemoprevention for lung cancer. The results of studies investigating selenium as a potential agent for lung cancer chemoprevention have been conflicting. In a secondary analysis of The Nutritional Prevention of Cancer Trial, a randomized, double-blind, placebo-controlled trial of over 1300 participants with a history of skin cancer [88], the incidence of lung cancer was reduced in individuals receiving selenium, although this difference was not statistically significant at a median follow-up of eight years [89]. A subgroup analysis showed that subjects with the lowest baseline selenium levels had the most significant decreased incidence, a finding that has been reported by others [90]. This agent was also studied in the Selenium and Vitamin E Cancer Prevention Trial (SELECT), a randomized double-blind placebo-controlled, multi-center study of selenium for prostate cancer prevention. In an analysis of lung cancer incidence as a secondary endpoint, there was no significant effect of selenomethionine alone or in combination with vitamin E on the development of lung cancer [91]. These studies suggest that there may be a differential primary chemopreventive effect dependent on baseline selenium levels, such that supplementation with selenium may reduce lung cancer risk in individuals with low baseline levels of selenium, but may increase lung cancer risk in patients with high baseline selenium levels. Based on the available evidence, selenium is not currently recommended for the primary chemoprevention of lung cancer.

#### 5.4.2. Anti-Inflammatories

Cyclooxygenase-2 (COX-2), an enzyme in the arachidonic acid cascade, is up-regulated in many tumors, including lung cancer. Non-steroidal anti-inflammatory drugs (NSAIDs) inhibit COX enzymes, and have been investigated as potential agents in the primary chemoprevention of lung cancer. One meta-analysis that included 19 studies of NSAIDs and lung-cancer risk suggests an overall benefit of regularly taking aspirin (but not other NSAIDs) for those at high risk of lung cancer [92], and a second analysis suggests that aspirin may decrease the 20-year risk of death from several cancers including lung adenocarcinoma [95]. In a prospective cohort study, NSAIDs were associated with a small reduced risk of lung cancer that was strongest for adenocarcinoma in men and in long-term former smokers [93]. The Women’s Health Study, the only randomized, placebo-controlled trial of aspirin, found that women taking low-dose aspirin had a borderline statistically-significant reduction in lung cancer risk [94].

Prostacyclin analogs, in addition to their anti-inflammatory effects, selectively increase peroxisome proliferator-activated receptor gamma (PPARγ) activity in non-small cell lung cancer [113]. In NSCLC cell lines, activation of PPARγ inhibits cell growth [114], and in murine models, PPARγ over-expression prevents lung cancer. Thiazolidinediones are oral PPARγ agonists used to treat diabetes. In a study of over 87,000 veterans treated for diabetes, patients on thiazolidinediones had 33% lower incidence of lung cancer compared to those on other medications for diabetes management [96]. A recent meta-analysis confirms a small, but significantly decreased risk of lung cancers in diabetics treated with thiazolidinediones [97]. An ongoing trial at the Denver Veterans Affairs Medical Center is evaluating the effect of the PPARγ agonist, pioglitazone, on lung cancer incidence in high-risk current and former smokers [98].

### 5.5. Secondary Chemoprevention

#### 5.5.1. Antioxidants

Several randomized trials studied the effects of retinoids given alone [99,100,101,102] or in combination with beta-carotene [103] on bronchial metaplasia seen in biopsy specimens or on sputum atypia. These trials did not demonstrate a statistically significant benefit from retinoid chemoprevention. Anethole dithiolethione (ADT), another antioxidant, has shown some evidence of slowing the progression of neoplastic changes. In a randomized placebo-controlled trial of 112 current or former smokers with at least one biopsy-proven area of bronchial dysplasia, there was less frequent progression of dysplasia in subjects treated with ADT, although dysplasia was not reversed in smokers [104]. 

#### 5.5.2. Anti-Inflammatories

The selective cyclooxygenase-2 (COX-2) inhibitor celecoxib was studied in two randomized trials in smokers, in which cellular proliferation and COX-2 expression levels were study endpoints [105,106]. In both studies, cellular proliferation in bronchial epithelium and BAL cells decreased after celecoxib treatment. Downstream in the cyclooxygenase pathway, the prostacyclin analogs have anti-inflammatory effects. Iloprost, a long-lasting oral prostacyclin analog inhibits lung tumorigenesis in carcinogen-exposed wild-type mice [115], and was studied in a national randomized, double-blinded placebo controlled trial in smokers or former smokers with biopsy-proven bronchial dysplasia [107]. The primary endpoint of the study was endobronchial histology. Six months of oral iloprost was found to significantly decrease endobronchial dysplasia at follow-up bronchoscopy in former smokers. Additional studies of the effectiveness of iloprost and celecoxib in the secondary chemoprevention of lung cancer and lung cancer mortality are needed. Early stage clinical trials have also examined the effect of inhaled budesonide in CT-detected lung nodules. While a phase IIb trial of inhaled budesonide showed no effect on the incidence or regression of dysplastic airway lesions, a decrease in the size of CT-detected nodules was observed [116]. A subsequent phase IIb trial to further investigate this observation showed no significant difference in nodule size following 1 year of budesonide therapy, but a trend towards regression of non-solid and partially solid nodules [117].

#### 5.5.3. PI3K/AKT/mTOR Pathway Inhibitors

Phosphatidylinositol 3-kinases (PI3Ks) regulate cell growth and survival through activation of protein kinase B (PKB or AKT) in the PI3K/AKT/mTOR pathway. PI3K is a well-recognized oncogenic pathway, with higher levels of pathway activity associated with several malignancies including lung cancer. Myo-inositol (*cis*-1,2,3,5-*trans*-4,6-cyclohexanehexol) is a well-tolerated inhibitor of the PI3K pathway. A clinical study of 10 smokers with bronchial dysplasia demonstrated a significant rate of regression of the dysplastic lesions following treatment with myo-inositol [108]. Interestingly, the PI3K pathway was increased in the cytologically normal bronchial airway of individuals with pre-cancerous dysplasic lesions, and reversed in those participants who responded to myo-inositol [109]. These findings suggest that genomic markers of oncogenic pathway activation might have utility for targeting secondary chemoprevention strategies to specific subgroups of patients.

### 5.6. Tertiary Chemoprevention: Antioxidants

Trials to prevent the development of a second lung cancer have evaluated agents such as retinyl palmitate, isotretinoin, or the combination of retinyl palmitate plus *N*-acetylcysteine. Chemoprevention efforts in this setting have not demonstrated an improvement in survival. In a phase III randomized placebo-controlled trial of the retinoid isotretinoin in patients with resected Stage I non-small cell lung cancer, there was no statistically significant difference in time to second primary tumors, recurrence rates, or mortality [110]. In the EUROSCAN trial, patients with either lung cancer or head and neck cancer were randomly assigned to treatment with retinyl palmitate, *N*-acetylcysteine, both, or placebo for two years [111]. These agents also did not impact the incidence of second primary tumors or in overall and event-free survival. 

Selenium has also been studied in the tertiary prevention of lung cancer. In a randomized, double-blind, placebo-controlled multi-center trial of selenium supplementation on the incidence of second primary tumors in individuals surgically resected Stage I non-small cell lung cancer, selenium conferred no benefit over placebo in the prevention of secondary primary tumors in patients with resected NSCLC [112]. The interaction between selenium level and efficacy in tertiary prevention remains to be determined, and it is unclear if selenium benefits only those with low baseline levels or if very high levels of selenium increase the risk of secondary primary lung cancers.

In these three tertiary chemoprevention trials, the lowest rate of second primary tumors was in never-smokers, followed by former smokers. No favorable effect of supplements (including selenium and retinoids) was observed in current smokers, suggesting that lung cancer chemopreventive approaches may have the greatest likelihood of success in the absence of ongoing tobacco-driven carcinogenesis [112]. 

## 6. Conclusions

### 6.1. The Future of Lung Cancer Prevention, Screening, and Early Detection

A multi-faceted approach combining smoking cessation and prevention, early lung cancer detection, and targeted chemoprevention is urgently needed to improve mortality from lung cancer. Since there are approximately 45 million former smokers in the United States and more than 1.2 billion smokers worldwide, tobacco use must be the first target, since smoking prevention and cessation are the most effective ways to prevent lung cancer. Identifying which former and current smokers are at highest risk for developing lung cancer is the next most important step in any screening, early detection, or chemoprevention program. Several groups have used genomic studies in an attempt to identify a subset of smokers who are at highest risk of developing lung cancer. Ideally, these approaches must be integrated with clinical early detection and screening strategies such as low-dose computer tomography (LDCT) scanning to effectively differentiate benign from malignant lung nodules, and to establish therapeutically exploitable differences between normal, precancerous, and malignant cells. A combined approach that integrates molecular biomarkers with CT characteristics (ex: volume doubling time, nodule size) may further help distinguish biologically aggressive lung cancers from indolent lesions. Since molecular, genetic, and epigenetic abnormalities precede morphological changes in the bronchi and alveoli, molecular biomarkers may help identify a group of high-risk patients who would most benefit from LDCT screening. Genetic abnormalities can be detected from respiratory cells from sputum, bronchial airway samples, and blood. Gene expression profiles generated from those specimens offer a wide area of investigation for biomarker development, though to be clinically applicable, biomarkers must be specific, cost-effective, and efficient.

The availability of agents that might be effective in reversing the pre-malignant changes in airway epithelial cells has driven the search for biomarkers that identify individuals at highest risk for developing lung cancer. Chemopreventive agents are increasingly selected for further development based on the biologic mechanisms underlying lung carcinogenesis. Given the long latency between progression from pre-malignancy to overt lung cancer, consensus groups in the United State and Europe have suggested that it is essential to identify earlier end-points in the study and development of chemopreventive agents [118,119]. 

### 6.2. Intermediate Markers of Disease Risk as Potential Targets for Personalized Chemoprevention

Serial tissue sampling could be used to measure biomarkers that reflect the clinical effectiveness of chemopreventative agents. As an example of this approach, Mascaux and colleagues used biospecimens from the iloprost lung cancer chemoprevention trial to investigate whether miRNAs could serve as predictive biomarkers or intermediate endpoints of response to iloprost [120]. The expression levels of 14 miRNAs were measured in matched bronchial biopsies before and after treatment with iloprost or placebo. While no predictive biomarkers of iloprost response were identified, down-regulation of miR-34c expression correlated with histological response to iloprost in serial biopsy specimens. An alternative approach to studying chemoprevention is to develop biomarkers that reflect the activity of oncogenic pathways and monitor these signatures longitudinally. Gustafson and colleagues investigated the behavior of a signature of PI3K pathway activation in cytologically normal bronchial airway cells obtained from smokers with and without distal lung cancer, and smokers with pre-cancerous airway lesions. They observed increased PI3K pathway activation in both the normal bronchial airway cells from patients with distal lung cancers, and in the normal bronchial airway cells from smokers with dysplastic airway lesions. Furthermore, high-risk smokers who had significant regression in dysplasia when treated with the PI3K pathway inhibitor, myo-inositol, demonstrated decreased PI3K activity in follow-up sampling of normal bronchial airway cells [108]. These findings suggest that a subset of individuals have abnormal activation of the PI3K pathway in bronchial airway epithelial cells that may contribute to lung cancer development, and may serve as an early, quantitative marker for response to targeted chemoprevention. 

While chemoprevention for lung cancer is not currently recommended, it remains a promising strategy to potentially reduce lung cancer mortality. Studies using biomarker and pathway-driven approaches aimed at identifying the subsets of patients most likely to respond to specific agents can serve as models for assessing novel molecular targets and chemopreventive agents. It is also possible that combination-targeted therapy directed at multiple oncogenic pathways may ultimately prove more effective than single agents alone. Integrating clinical markers of lung cancer risk with molecular biomarkers may also help improve early lung cancer detection through the development of comprehensive risk-assessment and early-detection models. Incorporating these biomarkers and integrating clinico-genomic models using surrogate tissue sampling into large-scale clinical studies promises to improve our ability to diagnose, treat, and prevent lung cancer and thereby improve mortality from this disease.

## References

[1] Siegel R., Naishadham D., Jemal A. (2013). Cancer statistics, 2013. CA Cancer J. Clin..

[2] Yabroff K.R., Lamont E.B., Mariotto A., Warren J.L., Topor M., Meekins A., Brown M.L. (2008). Cost of care for elderly cancer patients in the United States. J. Natl. Cancer Inst..

[3] Howlader N., Noone A.M., Krapcho M., Garshell J., Neyman N., Altekruse S.F., Kosary C.L., Yu M., Ruhl J., Tatalovich Z. SEER cancer statistics review (CSR), 1975–2010. http://seer.cancer.gov/csr/1975_2010/sections.html.

[4] Humphrey L.L., Deffebach M., Pappas M., Baumann C., Artis K., Mitchell J.P., Zakher B., Fu R., Slatore C.G. (2013). Screening for lung cancer with low-dose computed tomography: A systematic review to update the US Preventive services task force recommendation. Ann. Intern. Med..

[5] Wakelee H.A., Chang E.T., Gomez S.L., Keegan T.H., Feskanich D., Clarke C.A., Holmberg L., Yong L.C., Kolonel L.N., Gould M.K. (2007). Lung cancer incidence in never smokers. J. Clin. Oncol..

[6] Thu K.L., Vucic E.A., Chari R., Zhang W., Lockwood W.W., English J.C., Fu R., Wang P., Feng Z., MacAulay C.E. (2012). Lung adenocarcinoma of never smokers and smokers harbor differential regions of genetic alteration and exhibit different levels of genomic instability. PLoS One.

[7] Bach P.B., Kattan M.W., Thornquist M.D., Kris M.G., Tate R.C., Barnett M.J., Hsieh L.J., Begg C.B. (2003). Variations in lung cancer risk among smokers. J. Natl. Cancer Inst..

[8] Cronin K.A., Gail M.H., Zou Z., Bach P.B., Virtamo J., Albanes D. (2006). Validation of a model of lung cancer risk prediction among smokers. J. Natl. Cancer Inst..

[9] Spitz M.R., Hong W.K., Amos C.I., Wu X., Schabath M.B., Dong Q., Shete S., Etzel C.J. (2007). A risk model for prediction of lung cancer. J. Natl. Cancer Inst..

[10] Cassidy A., Myles J.P., van Tongeren M., Page R.D., Liloglou T., Duffy S.W., Field J.K. (2008). The LLP risk model: An individual risk prediction model for lung cancer. Br. J. Cancer.

[11] D’Amelio A.M., Cassidy A., Asomaning K., Raji O.Y., Duffy S.W., Field J.K., Spitz M.R., Christiani D., Etzel C.J. (2010). Comparison of discriminatory power and accuracy of three lung cancer risk models. Cancer Prev. Res. (Phila).

[12] Iyen-Omofoman B., Tata L.J., Baldwin D.R., Smith C.J.P., Hubbard R.B. (2013). Using socio-demographic and early clinical features in general practice to identify people with lung cancer earlier. Thorax.

[13] Maisonneuve P., Bagnardi V., Bellomi M., Spaggiari L., Pelosi G., Rampinelli C., Bertolotti R., Rotmensz N., Field J.K., Decensi A. (2011). Lung cancer risk prediction to select smokers for screening CT—A model based on the Italian COSMOS trial. Cancer Prev. Res. (Phila).

[14] Thun M.J., Henley S.J., Calle E.E. (2002). Tobacco use and cancer: An epidemiologic perspective for geneticists. Oncogene.

[15] Cooper D.N. (2005). The Molecular Genetics of Lung Cancer.

[16] Zienolddiny S., Campa D., Lind H., Ryberg D., Skaug V., Stangeland L., Phillips D.H., Canzian F., Haugen A. (2006). Polymorphisms of DNA repair genes and risk of non-small cell lung cancer. Carcinogenesis.

[17] Young R.P., Hopkins R.J., Hay B.A., Epton M.J., Mills G.D., Black P.N., Gardner H.D., Sullivan R., Gamble G.D. (2009). Lung cancer susceptibility model based on age, family history and genetic variants. PLoS One.

[18] Spitz M.R., Etzel C.J., Dong Q., Amos C.I., Wei Q., Wu X., Hong W.K. (2008). An expanded risk prediction model for lung cancer. Cancer Prev. Res. (Phila).

[19] Patz E.F., Goodman P.C., Bepler G. (2000). Screening for lung cancer. N. Engl. J. Med..

[20] Brett G.Z. (1969). Earlier diagnosis and survival in lung cancer. Br. Med. J..

[21] Oken M.M., Hocking W.G., Kvale P.A., Andriole G.L., Buys S.S., Church T.R., Crawford E.D., Fouad M.N., Isaacs C., Reding D.J. (2011). Screening by chest radiograph and lung cancer mortality: The Prostate, Lung, Colorectal, and Ovarian (PLCO) randomized trial. JAMA.

[22] Marcus P.M., Bergstralh E.J., Fagerstrom R.M., Williams D.E., Fontana R., Taylor W.F., Prorok P.C. (2000). Lung cancer mortality in the Mayo Lung Project: Impact of extended follow-up. J. Natl. Cancer Inst..

[23] Jett J.R., Midthun D.E. (2004). Screening for lung cancer: Current status and future directions: Thomas A. Neff lecture. Chest.

[24] Manser R., Lethaby A., Irving L.B., Stone C., Byrnes G., Abramson M.J., Campbell D. (2013). Screening for lung cancer. Cochrane Database Syst. Rev..

[25] Aberle D.R., Adams A.M., Berg C.D., Black W.C., Clapp J.D., Fagerstrom R.M., Gareen I.F., Gatsonis C., Marcus P.M. (2011). Trial research team reduced lung-cancer mortality with low-dose computed tomographic screening. N. Engl. J. Med..

[26] Moyer V.A. (2013). Screening for Lung Cancer: U.S. preventive services task force recommendation statement. Ann. Intern. Med..

[27] Van Iersel C.A., de Koning H.J., Draisma G., Mali W.P.T.M., Scholten E.T., Nackaerts K., Prokop M., Habbema J.D.F., Oudkerk M., van Klaveren R.J. (2007). Risk-based selection from the general population in a screening trial: Selection criteria, recruitment and power for the Dutch-Belgian randomised lung cancer multi-slice CT screening trial (NELSON). Int. J. Cancer.

[28] Heuvelmans M.A., Oudkerk M., de Bock G.H., de Koning H.J., Xie X., van Ooijen P.M.A., Greuter M.J.W., de Jong P.A., Groen H.J.M., Vliegenthart R. (2013). Optimisation of volume-doubling time cutoff for fast-growing lung nodules in CT lung cancer screening reduces false-positive referrals. Eur. Radiol..

[29] Saghir Z., Dirksen A., Ashraf H., Bach K.S., Brodersen J., Clementsen P.F., Døssing M., Hansen H., Kofoed K.F., Larsen K.R. (2012). CT screening for lung cancer brings forward early disease. The randomised Danish Lung Cancer Screening Trial: Status after five annual screening rounds with low-dose CT. Thorax.

[30] Nackaerts K., Vansteenkiste J. (2009). Low-dose CT screening for lung cancer: Trial and error?. J. Thorac. Oncol..

[31] Van den Bergh K.A.M., Essink-Bot M.-L., Bunge E.M., Scholten E.T., Prokop M., van Iersel C.A., van Klaveren R.J., de Koning H.J. (2008). Impact of computed tomography screening for lung cancer on participants in a randomized controlled trial (NELSON trial). Cancer.

[32] Van den Bergh K.A.M., Essink-Bot M.L., Borsboom G.J.J.M., Scholten E.T., van Klaveren R.J., de Koning H.J. (2011). Long-term effects of lung cancer computed tomography screening on health-related quality of life: The NELSON trial. Eur. Respir. J..

[33] Braithwaite D., Zhu W., Hubbard R.A., O’Meara E.S., Miglioretti D.L., Geller B., Dittus K., Moore D., Wernli K.J., Mandelblatt J. (2013). Screening outcomes in older US women undergoing multiple mammograms in community practice: Does interval, age, or comorbidity score affect tumor characteristics or false positive rates?. J. Natl. Cancer Inst..

[34] Kovalchik S.A., Tammemagi M., Berg C.D., Caporaso N.E., Riley T.L., Korch M., Silvestri G.A., Chaturvedi A.K., Katki H.A. (2013). Targeting of low-dose CT screening according to the risk of lung-cancer death. N. Engl. J. Med..

[35] Mettler F.A., Huda W., Yoshizumi T.T., Mahesh M. (2008). Effective doses in radiology and diagnostic nuclear medicine: A catalog. Radiology.

[36] Bach P.B., Mirkin J.N., Oliver T.K., Azzoli C.G., Berry D.A., Brawley O.W., Byers T., Colditz G.A., Gould M.K., Jett J.R. (2012). Benefits and harms of CT screening for lung cancer: A systematic review. JAMA.

[37] Tokarskaya Z.B., Scott B.R., Zhuntova G.V., Okladnikova N.D., Belyaeva Z.D., Khokhryakov V.F., Schöllnberger H., Vasilenko E.K. (2002). Interaction of radiation and smoking in lung cancer induction among workers at the Mayak nuclear enterprise. Health Phys..

[38] Silvestri G.A. (2011). Screening for lung cancer: It works, but does it really work?. Ann. Intern. Med..

[39] Patz E.F., Pinsky P., Gatsonis C., Sicks J.D., Kramer B.S., Tammemägi M.C., Chiles C., Black W.C., Aberle D.R. (2013). Overdiagnosis in low-dose computed tomography screening for lung cancer. JAMA Intern. Med..

[40] Yu L., Todd N.W., Xing L., Xie Y., Zhang H., Liu Z., Fang H., Zhang J., Katz R.L., Jiang F. (2010). Early detection of lung adenocarcinoma in sputum by a panel of microRNA markers. Int. J. Cancer.

[41] Franklin W.A., Gazdar A.F., Haney J., Wistuba I.I., LaRosa F.G., Kennedy T., Ritchey D.M., Miller Y.E. (1997). Widely dispersed p53 mutation in respiratory epithelium. A novel mechanism for field carcinogenesis. J. Clin. Investig..

[42] Wistuba I.I., Lam S., Behrens C., Virmani A.K., Fong K.M., LeRiche J., Samet J.M., Srivastava S., Minna J.D., Gazdar A.F. (1997). Molcular damage in the bronchial epithelium of current and former smokers. J. Natl. Cancer Inst..

[43] Mao L., Lee J.S., Kurie J.M., Fan Y.H., Lippman S.M., Lee J.J., Ro J.Y., Broxson A., Yu R., Morice R.C. (1997). Clonal genetic alterations in the lungs of current and former smokers. J. Natl. Cancer Inst..

[44] Tang X., Shigematsu H., Bekele B.N., Roth J.A., Minna J.D., Hong W.K., Gazdar A.F., Wistuba I.I. (2005). EGFR tyrosine kinase domain mutations are detected in histologically normal respiratory epithelium in lung cancer patients. Cancer Res..

[45] Powell C.A., Klares S., O’Connor G., Brody J.S. (1999). Loss of heterozygosity in epithelial cells obtained by bronchial brushing: Clinical utility in lung cancer. Clin. Cancer Res..

[46] Spira A., Beane J.E., Shah V., Steiling K., Liu G., Schembri F., Gilman S., Dumas Y.-M., Calner P., Sebastiani P. (2007). Airway epithelial gene expression in the diagnostic evaluation of smokers with suspect lung cancer. Nat. Med..

[47] Beane J., Sebastiani P., Whitfield T.H., Steiling K., Dumas Y.-M., Lenburg M.E., Spira A. (2008). A prediction model for lung cancer diagnosis that integrates genomic and clinical features. Cancer Prev. Res. (Phila).

[48] Li Q.K., Shah P., Li Y., Aiyetan P.O., Chen J., Yung R., Molena D., Gabrielson E., Askin F., Chan D.W. (2013). Glycoproteomic analysis of bronchoalveolar lavage (BAL) fluid identifies tumor-associated glycoproteins from lung adenocarcinoma. J. Proteome Res..

[49] Hassanein M., Callison J.C., Callaway-Lane C., Aldrich M.C., Grogan E.L., Massion P.P. (2012). The state of molecular biomarkers for the early detection of lung cancer. Cancer Prev. Res. (Phila).

[50] Spira A., Beane J., Shah V., Liu G., Schembri F., Yang X., Palma J., Brody J.S. (2004). Effects of cigarette smoke on the human airway epithelial cell transcriptome. Proc. Natl. Acad. Sci. USA.

[51] Steiling K., Kadar A.Y., Bergerat A., Flanigon J., Sridhar S., Shah V., Ahman R.Q., Brody J.S., Lenburg M.E., Steffen M. (2009). Comparison of proteomic and transcriptomic profiles in the bronchial airway of current and never smokers. PLoS One.

[52] Sridhar S., Schembri F., Zeskind J., Shah V., Gustafson A.M., Steiling K., Liu G.,  Dumas Y.-M., Zhang X., Brody J.S. (9). Smoking-induced gene expression changes in the bronchial airway are reflected in nasal and buccal epithelium. BMC Genomics.

[53] Zhang X., Sebastiani P., Liu G., Schembri F., Zhang X., Dumas Y.M., Langer E.M., Alekseyev Y., O’Connor G.T., Brooks D.R. (2010). Similarities and differences between smoking-related gene expression in nasal and bronchial epithelium. Physiol. Genomics.

[54] Boyle J.O., Gümüs Z.H., Kacker A., Choksi V.L., Bocker J.M., Zhou X.K., Yantiss R.K., Hughes D.B., Du B., Judson B.L. (2010). Effects of cigarette smoke on the human oral mucosal transcriptome. Cancer Prev. Res. (Phila).

[55] Flehinger B.J., Melamed M.R., Zaman M.B., Heelan R.T., Perchick W.B., Martini N. (1984). Early lung cancer detection: Results of the initial (prevalence) radiologic and cytologic screening in the Memorial Sloan-Kettering study. Am. Rev. Respir. Dis..

[56] Mao L., Hruban R.H., Boyle J.O., Tockman M., Sidransky D. (1994). Detection of oncogene mutations in sputum precedes diagnosis of lung cancer. Cancer Res..

[57] Li R., Todd N.W., Qiu Q., Fan T., Zhao R.Y., Rodgers W.H., Fang H.-B., Katz R.L., Stass S.A., Jiang F. (2007). Genetic deletions in sputum as diagnostic markers for early detection of stage I non-small cell lung cancer. Clin. Cancer Res..

[58] Belinsky S.A., Palmisano W.A., Gilliland F.D., Crooks L.A., Divine K.K., Winters S.A., Grimes M.J., Harms H.J., Tellez C.S., Smith T.M. (2002). Aberrant promoter methylation in bronchial epithelium and sputum from current and former smokers. Cancer Res..

[59] Palmisano W.A., Divine K.K., Saccomanno G., Gilliland F.D., Baylin S.B., Herman J.G., Belinsky S.A. (2000). Predicting lung cancer by detecting aberrant promoter methylation in sputum. Cancer Res..

[60] Diamandis E.P., Goodglick L., Planque C., Thornquist M.D. (2011). Pentraxin-3 is a novel biomarker of lung carcinoma. Clin. Cancer Res..

[61] Ajona D., Pajares M.J., Corrales L., Perez-Gracia J.L., Agorreta J., Lozano M.D., Torre W., Massion P.P., de-Torres J.P., Jantus-Lewintre E. (2013). Investigation of complement activation product c4d as a diagnostic and prognostic biomarker for lung cancer. J. Natl. Cancer Inst..

[62] Boeri M., Verri C., Conte D., Roz L., Modena P., Facchinetti F., Calabrò E., Croce C.M., Pastorino U., Sozzi G. (2011). MicroRNA signatures in tissues and plasma predict development and prognosis of computed tomography detected lung cancer. Proc. Natl. Acad. Sci. USA.

[63] Bianchi F., Nicassio F., Marzi M., Belloni E., Dall’olio V., Bernard L., Pelosi G., Maisonneuve P., Veronesi G., di Fiore P.P. (2011). A serum circulating miRNA diagnostic test to identify asymptomatic high-risk individuals with early stage lung cancer. EMBO Mol. Med..

[64] Bibikova M., Lin Z., Zhou L., Chudin E., Garcia E.W., Wu B., Doucet D., Thomas N.J., Wang Y., Vollmer E. (2006). High-throughput DNA methylation profiling using universal bead arrays. Genome Res..

[65] Wilson I.M., Davies J.J., Weber M., Brown C.J., Alvarez C.E., MacAulay C., Schübeler D., Lam W.L. (2006). Epigenomics: Mapping the methylome. Cell Cycle.

[66] Ostroff R.M., Bigbee W.L., Franklin W., Gold L., Mehan M., Miller Y.E., Pass H.I., Rom W.N., Siegfried J.M., Stewart A. (2010). Unlocking biomarker discovery: Large scale application of aptamer proteomic technology for early detection of lung cancer. PLoS One.

[67] Tanaka F., Yoneda K., Kondo N., Hashimoto M., Takuwa T., Matsumoto S., Okumura Y., Rahman S., Tsubota N., Tsujimura T. (2009). Circulating tumor cell as a diagnostic marker in primary lung cancer. Clin. Cancer Res..

[68] Yildiz P.B., Shyr Y., Rahman J.S.M., Wardwell N.R., Zimmerman L.J., Shakhtour B., Gray W.H., Chen S., Li M., Roder H. (2007). Diagnostic accuracy of MALDI mass spectrometric analysis of unfractionated serum in lung cancer. J. Thorac. Oncol..

[69] Pecot C.V., Li M., Zhang X.J., Rajanbabu R., Calitri C., Bungum A., Jett J.R., Putnam J.B., Callaway-Lane C., Deppen S. (2012). Added value of a serum proteomic signature in the diagnostic evaluation of lung nodules. Cancer Epidemiol. Biomark. Prev..

[70] Li X., Hayward C., Fong P.-Y., Dominguez M., Hunsucker S.W., Lee L.W., McLean M., Law S., Butler H., Schirm M. (2013). A blood-based proteomic classifier for the molecular characterization of pulmonary nodules. Sci. Transl. Med..

[71] Shopland D.R. (1995). Tobacco use and its contribution to early cancer mortality with a special emphasis on cigarette smoking. Environ. Health Perspect..

[72] King B., Dube S., Kaufmann R., Shaw L., Pechacek T. (2011). Vital signs: Current cigarette smoking among adults aged ≥18 Years—United States, 2005–2010. Morb. Mortal. Wkly. Rep..

[73] Anthonisen N.R., Skeans M.A., Wise R.A., Manfreda J., Kanner R.E., Connett J.E. (2005). The effects of a smoking cessation intervention on 14.5-year mortality: A randomized clinical trial. Ann. Intern. Med..

[74] Villanti A.C., Jiang Y., Abrams D.B., Pyenson B.S. (2013). A cost-utility analysis of lung cancer screening and the additional benefits of incorporating smoking cessation interventions. PLoS One.

[75] Sporn M.B. (1976). Approaches to prevention of epithelial cancer during the preneoplastic period. Cancer Res..

[76] Hirsch F.R., Lippman S.M. (2005). Advances in the biology of lung cancer chemoprevention. J. Clin. Oncol..

[77] Kelloff G.J., Lippman S.M., Dannenberg A.J., Sigman C.C., Pearce H.L., Reid B.J., Szabo E., Jordan V.C., Spitz M.R., Mills G.B. (2006). Progress in chemoprevention drug development: The promise of molecular biomarkers for prevention of intraepithelial neoplasia and cancer—A plan to move forward. Clin. Cancer Res..

[78] Khuri F.R., Cohen V. (2004). Molecularly targeted approaches to the chemoprevention of lung cancer. Clin. Cancer Res..

[79] Keith R.L. (2009). Chemoprevention of lung cancer. Proc. Am. Thorac. Soc..

[80] Greenberg A.K., Tsay J.-C., Tchou-Wong K.-M., Jorgensen A., Rom W.N. (2013). Chemoprevention of lung cancer: Prospects and disappointments in human clinical trials. Cancers.

[81] Keith R.L., Miller Y.E. (2013). Lung cancer chemoprevention: Current status and future prospects. Nat. Rev. Clin. Oncol..

[82] The α-Tocopherol, β carotene cancer prevention study group (1994). The effect of vitamin E and beta carotene on the incidence of lung cancer and other cancers in male smokers. N. Engl. J. Med..

[83] Lee I.M., Cook N.R., Manson J.E., Buring J.E., Hennekens C.H. (1999). Beta-carotene supplementation and incidence of cancer and cardiovascular disease: The Women’s Health Study. J. Natl. Cancer Inst..

[84] Hennekens C.H., Buring J.E., Manson J.E., Stampfer M., Rosner B., Cook N.R., Belanger C., LaMotte F., Gaziano J.M., Ridker P.M. (1996). Lack of effect of long-term supplementation with beta carotene on the incidence of malignant neoplasms and cardiovascular disease. N. Engl. J. Med..

[85] Albanes D., Heinonen O.P., Taylor P.R., Virtamo J., Edwards B.K., Rautalahti M., Hartman A.M., Palmgren J., Freedman L.S., Haapakoski J. (1996). α-Tocopherol and beta-carotene supplements and lung cancer incidence in the α-tocopherol, beta-carotene cancer prevention study: Effects of base-line characteristics and study compliance. J. Natl. Cancer Inst..

[86] Virtamo J., Pietinen P., Huttunen J.K., Korhonen P., Malila N., Virtanen M.J., Albanes D., Taylor P.R., Albert P., ATBC Study Group (2003). Incidence of cancer and mortality following alpha-tocopherol and beta-carotene supplementation: A postintervention follow-up. JAMA.

[87] Omenn G.S., Goodman G.E., Thornquist M.D., Balmes J., Cullen M.R., Glass A., Keogh J.P., Meyskens F.L., Valanis B., Williams J.H. (1996). Effects of a combination of beta carotene and vitamin A on lung cancer and cardiovascular disease. N. Engl. J. Med..

[88] Clark L.C., Combs G.F., Turnbull B.W., Slate E.H., Chalker D.K., Chow J., Davis L.S., Glover R.A., Graham G.F. (1996). Effects of selenium supplementation for cancer prevention in patients with carcinoma of the skin. A randomized controlled trial. Nutritional Prevention of Cancer Study Group. JAMA.

[89] Reid M.E., Duffield-Lillico A.J., Garland L., Turnbull B.W., Clark L.C., Marshall J.R. (2002). Selenium supplementation and lung cancer incidence: An update of the nutritional prevention of cancer trial. Cancer Epidemiol. Biomark. Prev..

[90] Fleet J.C. (1997). Dietary selenium repletion may reduce cancer incidence in people at high risk who live in areas with low soil selenium. Nutr. Rev..

[91] Lippman S.M., Klein E.A., Goodman P.J., Lucia M.S., Thompson I.M., Ford L.G., Parnes H.L., Minasian L.M., Gaziano J.M. (2009). Effect of selenium and vitamin E on risk of prostate cancer and other cancers: The Selenium and Vitamin E Cancer Prevention Trial (SELECT). JAMA.

[92] Xu J., Yin Z., Gao W., Liu L., Wang R., Huang P., Yin Y., Liu P., Yu R., Shu Y. (2012). Meta-analysis on the association between nonsteroidal anti-inflammatory drug use and lung cancer risk. Clin. Lung Cancer.

[93] Slatore C.G., Au D.H., Littman A.J., Satia J.A., White E. (2009). Association of nonsteroidal anti-inflammatory drugs with lung cancer: Results from a large cohort study. Cancer Epidemiol. Biomark. Prev..

[94] Cook N.R., Lee I.-M., Gaziano J.M., Gordon D., Ridker P.M., Manson J.E., Hennekens C.H., Buring J.E. (2005). Low-dose aspirin in the primary prevention of cancer: The Women’s Health Study: A randomized controlled trial. JAMA.

[95] Rothwell P.M., Fowkes F.G.R., Belch J.F.F., Ogawa H., Warlow C.P., Meade T.W. (2011). Effect of daily aspirin on long-term risk of death due to cancer: Analysis of individual patient data from randomised trials. Lancet.

[96] Govindarajan R., Ratnasinghe L., Simmons D.L., Siegel E.R., Midathada M.V., Kim L., Kim P.J., Owens R.J., Lang N.P. (2007). Thiazolidinediones and the risk of lung, prostate, and colon cancer in patients with diabetes. J. Clin. Oncol..

[97] Colmers I.N., Bowker S.L., Johnson J.A. (2012). Thiazolidinedione use and cancer incidence in type 2 diabetes: A systematic review and meta-analysis. Diabetes Metab..

[98] Pioglitazone for Lung Cancer Chemoprevention. http://clinicaltrials.gov/show/NCT00780234.

[99] Lee J.S., Lippman S.M., Benner S.E., Lee J.J., Ro J.Y., Lukeman J.M., Morice R.C., Peters E.J., Pang A.C., Fritsche H.A. (1994). Randomized placebo-controlled trial of isotretinoin in chemoprevention of bronchial squamous metaplasia. J. Clin. Oncol..

[100] Kelly K., Kittelson J., Franklin W.A., Kennedy T.C., Klein C.E., Keith R.L., Dempsey E.C., Lewis M., Jackson M.K., Hirsch F.R. (2009). A randomized phase II chemoprevention trial of 13-CIS retinoic acid with or without alpha tocopherol or observation in subjects at high risk for lung cancer. Cancer Prev. Res. (Phila).

[101] Arnold A.M., Browman G.P., Levine M.N., D’Souza T., Johnstone B., Skingley P., Turner-Smith L., Cayco R., Booker L., Newhouse M. (1992). The effect of the synthetic retinoid etretinate on sputum cytology: Results from a randomised trial. Br. J. Cancer.

[102] Kurie J.M., Lee J.S., Khuri F.R., Mao L., Morice R.C., Lee J.J., Walsh G.L., Broxson A., Lippman S.M., Ro J.Y. (2000). *N*-(4-hydroxyphenyl)retinamide in the chemoprevention of squamous metaplasia and dysplasia of the bronchial epithelium. Clin. Cancer Res..

[103] McLarty J.W., Holiday D.B., Girard W.M., Yanagihara R.H., Kummet T.D., Greenberg S.D. (1995). Beta-Carotene, vitamin A, and lung cancer chemoprevention: Results of an intermediate endpoint study. Am. J. Clin. Nutr..

[104] Lam S., MacAulay C., le Riche J.C., Dyachkova Y., Coldman A., Guillaud M., Hawk E., Christen M.-O., Gazdar A.F. (2002). A randomized phase IIb trial of anethole dithiolethione in smokers with bronchial dysplasia. J. Natl. Cancer Inst..

[105] Mao J.T., Fishbein M.C., Adams B., Roth M.D., Goodglick L., Hong L., Burdick M., Strieter E.R.M., Holmes C., Tashkin D.P. (2006). Celecoxib decreases Ki-67 proliferative index in active smokers. Clin. Cancer Res..

[106] Kim E.S., Hong W.K., Lee J.J., Mao L., Morice R.C., Liu D.D., Jimenez C.A., Eapen G.A., Lotan R., Tang X. (2010). Biological activity of celecoxib in the bronchial epithelium of current and former smokers. Cancer Prev. Res. (Phila).

[107] Keith R.L., Blatchford P.J., Kittelson J., Minna J.D., Kelly K., Massion P.P., Franklin W.A., Mao J., Wilson D.O., Merrick D.T. (2011). Oral iloprost improves endobronchial dysplasia in former smokers. Cancer Prev. Res. (Phila).

[108] Lam S., McWilliams A., LeRiche J., MacAulay C., Wattenberg L., Szabo E. (2006). A phase I study of myo-inositol for lung cancer chemoprevention. Cancer Epidemiol. Biomark. Prev..

[109] Gustafson A.M., Soldi R., Anderlind C., Scholand M.B., Qian J., Zhang X., Cooper K., Walker D., McWilliams A., Liu G. (2010). Airway PI3K pathway activation is an early and reversible event in lung cancer development. Sci. Transl. Med..

[110] Lippman S.M., Lee J.J., Karp D.D., Vokes E.E., Benner S.E., Goodman G.E., Khuri F.R., Marks R., Winn R.J., Fry W. (2001). Randomized phase III intergroup trial of isotretinoin to prevent second primary tumors in stage I non-small-cell lung cancer. J. Natl. Cancer Inst..

[111] Van Zandwijk N., Dalesio O., Pastorino U., de Vries N., van Tinteren H. (2000). EUROSCAN, a randomized trial of vitamin A and *N*-acetylcysteine in patients with head and neck cancer or lung cancer. J. Natl. Cancer Inst..

[112] Karp D.D., Lee S.J., Keller S.M., Wright G.S., Aisner S., Belinsky S.A., Johnson D.H., Johnston M.R., Goodman G., Clamon G. (2013). Randomized, double-blind, placebo-controlled, phase III chemoprevention trial of selenium supplementation in patients with resected stage I non-small-cell lung cancer: ECOG 5597. J. Clin. Oncol..

[113] Wick M., Hurteau G., Dessev C., Chan D., Geraci M.W., Winn R.A., Heasley L.E., Nemenoff R.A. (2002). Peroxisome proliferator-activated receptor-gamma is a target of nonsteroidal anti-inflammatory drugs mediating cyclooxygenase-independent inhibition of lung cancer cell growth. Mol. Pharmacol..

[114] Bren-Mattison Y., van Putten V., Chan D., Winn R., Geraci M.W., Nemenoff R.A. (2005). Peroxisome proliferator-activated receptor-gamma (PPAR(gamma)) inhibits tumorigenesis by reversing the undifferentiated phenotype of metastatic non-small-cell lung cancer cells (NSCLC). Oncogene.

[115] Nemenoff R., Meyer A.M., Hudish T.M., Mozer A.B., Snee A., Narumiya S., Stearman R.S., Winn R.A., Weiser-Evans M., Geraci M.W. (2008). Prostacyclin prevents murine lung cancer independent of the membrane receptor by activation of peroxisomal proliferator—Activated receptor gamma. Cancer Prev. Res. (Phila).

[116] Lam S., leRiche J.C., McWilliams A., Macaulay C., Dyachkova Y., Szabo E., Mayo J., Schellenberg R., Coldman A., Hawk E. (2004). A randomized phase IIb trial of pulmicort turbuhaler (budesonide) in people with dysplasia of the bronchial epithelium. Clin. Cancer Res..

[117] Veronesi G., Szabo E., Decensi A., Guerrieri-Gonzaga A., Bellomi M., Radice D., Ferretti S., Pelosi G., Lazzeroni M., Serrano D. (2011). Randomized phase II trial of inhaled budesonide *versus* placebo in high-risk individuals with CT screen-detected lung nodules. Cancer Prev. Res. (Phila).

[118] Crowell J.A. (2005). The chemopreventive agent development research program in the Division of Cancer Prevention of the US National Cancer Institute: An overview. Eur. J. Cancer.

[119] Gerhauser C., Bartsch H., Crowell J., de Flora S., D’Incalci M., Dittrich C., Frank N., Mihich E., Steffen C., Tortora G. (2006). Development of novel cancer chemopreventive agents in Europe—neglected Cinderella or rising phoenix? A critical commentary. ESF Workshop on Cancer Chemoprevention, DKFZ, Heidelberg, September 18–20, 2005. Eur. J. Cancer.

[120] Mascaux C., Feser W.J., Lewis M.T., Barón A.E., Coldren C.D., Merrick D.T., Kennedy T.C., Eckelberger J.I., Rozeboom L.M., Franklin W.A. (2013). Endobronchial miRNAs as biomarkers in lung cancer chemoprevention. Cancer Prev. Res. (Phila).

